# Supplementary material for: Splice-Junction-Based Mapping of Alternative Isoforms in the Human Proteome
Source: Cell Rep. Author manuscript; Available in PMC 2020 Jan 15. (PMC6961840; doi:10.1016/j.celrep.2019.11.026)
Supplement: 3 [file NIHMS1546469-supplement-3.zip › DF2/PXD0010154/Lung-124-O76041-RPGIIVAPVLPGAYQQSHSQGYGY.pdf]

A

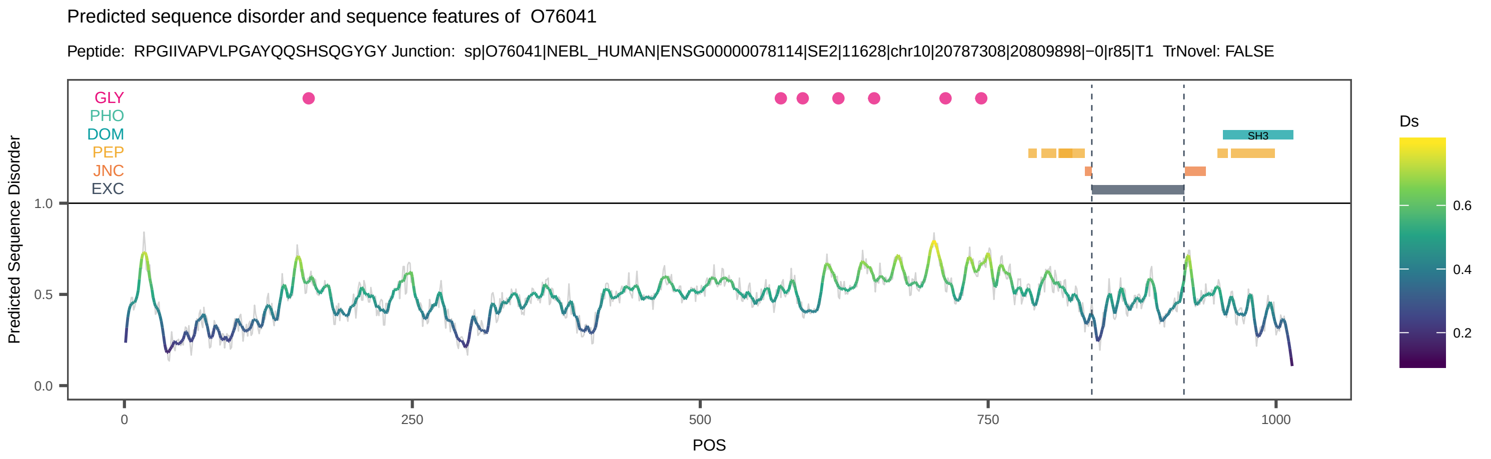

Predicted sequence disorder and sequence features of O76041
Peptide: RPGIIVAPVLPGAYQQSHSQGYGY Junction: sp|O76041|NEBL_HUMAN|ENSG00000078114|SE2|11628|chr10|20787308|20809898|−0|r85|T1 TrNovel: FALSE
GLY
PHO
DOM
PEP
JNC
EXC
SH3
Predicted Sequence Disorder
1.0
0.5
0.0
0
250
500
750
1000
POS
Ds
0.6
0.4
0.2

B

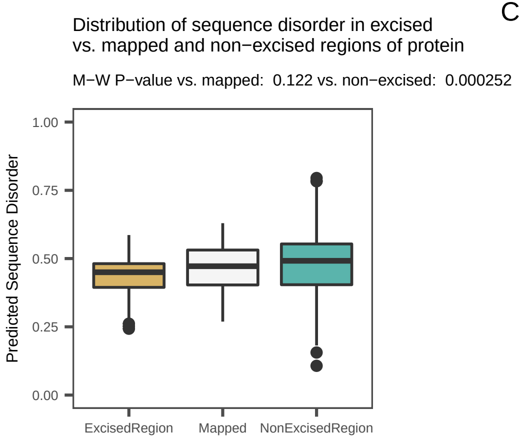

Distribution of sequence disorder in excised
vs. mapped and non−excised regions of protein
M−W P−value vs. mapped: 0.122 vs. non−excised: 0.000252
Predicted Sequence Disorder
1.00
0.75
0.50
0.25
0.00
ExcisedRegion
Mapped
NonExcisedRegion

C
